# Risk Charts Illustrating the 10-year Risk of Myocardial Infarction among Residents of Japanese Rural Communities: The JMS Cohort Study

**DOI:** 10.2188/jea.JE20080081

**Published:** 2009-03-19

**Authors:** Masatoshi Matsumoto, Shizukiyo Ishikawa, Kazunori Kayaba, Tadao Gotoh, Naoki Nago, Akizumi Tsutsumi, Eiji Kajii, the Jichi Medical School (JMS) Cohort Study Group

**Affiliations:** 1 Division of Community and Family Medicine, Center for Community Medicine, Jichi Medical University, Shimotsuke, Tochigi, Japan; 2 School of Health and Social Services, Saitama Prefectural University, Koshigaya, Saitama, Japan; 3 Wara National Health Insurance Clinic, Gujo, Gifu, Japan; 4 Community Medicine Education Center, Japan Association for Development of Community Medicine, Tokyo, Japan; 5 Occupational Health Training Center, University of Occupational and Environmental Health, Kitakyushu, Fukuoka, Tokyo

**Keywords:** myocardial infarction, blood pressure, cholesterol, smoking, diabetes, cohort study

## Abstract

**Background:**

Risk charts that depict the absolute risk of myocardial infarction (MI) for each combination of risk factors in individuals are convenient and beneficial tools for primary prevention of ischemic heart disease. Although risk charts have been developed using data from North American and European cardiovascular cohort studies, there is no such chart derived from cardiovascular incidence data obtained from the Japanese population.

**Methods and Results:**

We calculated and constructed risk charts that estimate the 10-year absolute risk of MI by using data from the Jichi Medical School (JMS) Cohort Study—a prospective cohort study which followed 12 490 participants in 12 Japanese rural communities for an average of 10.9 years. We identified 92 cases of a clinically-certified MI event. Color-coded risk charts were created by calculating the absolute risk associated with the following conventional cardiovascular risk factors: age, sex, smoking status, diabetes status, systolic blood pressure, and serum total cholesterol.

**Conclusions:**

In health education and clinical practice, particularly in rural communities, these charts should prove useful in understanding the risks of MI, without the need for cumbersome calculations. In addition, they can be expected to provide benefits by improving existing risk factors in individuals.

## INTRODUCTION

Ischemic heart disease is the leading cause of death in most Western countries.^1^ In Japan, although mortality from ischemic heart disease is much lower than that in Western countries, it is still a major cause of death.^1^^,^^2^ Risk factors known to have an impact on ischemic heart disease include age, sex, blood pressure, smoking, lipid profile, and diabetes.^3^ Recently, population groups with increased coronary risks, including individuals with hyperlipidemia and those with diabetes, have been growing rapidly in Japan. Thus, there is the potential for a commensurate increase in the incidence of ischemic heart disease in the near future. However, some cardiovascular cohort studies conducted in Japan have indicated that the incidence of coronary heart disease has remained steady for the last several decades, and that mortality has slightly decreased.^2^^,^^4^^,^^5^ It is hypothesized that this trend is due to recent improvements in treatment measures for coronary heart disease, and to improved risk factor management, including pervasive hypertension control and a decrease in the rate of tobacco use.^3^

For each individual with one or more risk factors, it is beneficial to know the probability of coronary heart disease. This information would enable the individual to appreciate the necessity of medical intervention and self-management of risk factors. For this purpose, several methods for calculating the absolute risk of coronary heart disease have been developed using data from large-scale cohort studies.^6^^,^^7^ Calculations using complicated mathematical formulae are cumbersome, however, and therefore not suitable for health education in communities and clinics. Risk charts that clearly illustrate the absolute risk of coronary heart disease for each combination of risk factors allow for improved public understanding and participation. Such charts have been developed using data from the Framingham study in the United States, the SCORE project in Europe, and the NIPPON DATA80 study in Japan, and are now available for use.^8^^–^^10^

However, there are problems if Japanese clinicians and healthcare workers attempt to utilize existing risk charts that are based on data from cohort studies in the United States and Europe. First, the incidences of coronary heart disease in these countries substantially differ from that observed in Japan. In a comparison of the results from the Framingham study with data obtained from a community-based cohort study in Japan, the white middle class population in the United States has a 5 to 6 times higher incidence of myocardial infarction (MI).^5^^,^^11^^,^^12^ Applying the Framingham-based risk chart to a Japanese population thus considerably overestimates the risk of coronary heart disease. Second, there may be differences in the degree of impact of each risk factor on coronary heart disease among different racial and cultural groups. To cite one example, diabetes and hypercholesterolemia are strong contributing factors to MI in white American populations, but they were not identified as risk factors for MI in some Japanese cohort studies.^4^^,^^5^^,^^11^^,^^13^^–^^15^

Current risk charts based on Japanese cohort studies are therefore needed. Charts developed using data from NIPPON DATA80 are now available in Japan. Although these charts were the first to use data from a Japanese population to assess cardiovascular risk, they are based on mortality data and the outcome measure was coronary death, not coronary incidence.^16^^,^^17^ These mortality data did not include non-fatal coronary events. Moreover, diagnoses in the NIPPON DATA80 were based on data from death certificates, and the accuracy of a diagnosis in such circumstances is always a concern . In order to estimate accurately an individual’s risk of coronary heart disease, risk charts based on incidence data from the Japanese population are needed.

The Jichi Medical School (JMS) Cohort Study is a multi-community prospective study that follows rural residents in Japan and monitors cardiovascular disease events.^18^^–^^20^ In this article we utilize data from the JMS Cohort Study to develop risk assessment charts for MI.

## METHODS

### Study population

The JMS Cohort Study began in 1992. Its primary objective was to clarify the relations between potential risk factors and cardiovascular disease in 12 rural districts in Japan.^18^ The baseline data of this cohort study were obtained between April 1992 and July 1995. If several sets of data were obtained for a single participant during that period, the first set was used as the baseline information. The baseline data were collected as part of a national mass-screening program. In Japan, mass screening for cardiovascular disease has been conducted since 1982, in accordance with the Health and Medical Service for the Aged Act of 1981. Local government offices in each community issued invitations to eligible residents for the mass screening, and personal invitations were also sent to all potential participants by mail. As a result, 12 490 participants were eligible (4913 males and 7577 females) in the adult age groups (19–93 years). The overall response rate among the 12 communities was 65.0%.

Among the possible 12 490 participants, 95 (0.8%) who did not sign the agreement to participate in the study, 7 (0.06%) who had no follow-up data, and 65 (0.5%) who had a past history of MI were excluded. The final study group eligible for analysis therefore comprised 12 323 participants (4829 men and 7494 women).

### Measurement of baseline variables

To synchronize the methods of data collection, we established a central committee composed of the chief medical officers from all the participating districts. This committee developed a detailed written methodology for data collection. Systolic blood pressure and diastolic blood pressure were measured with a fully-automated sphygmomanometer, the BP203RV-II (Nippon Colin, Komaki, Japan), placed on the right arm of a seated participant who had rested in a sitting position for 5 minutes before measurement. Information about medical history and lifestyle was gathered by means of a written questionnaire.

Blood samples were drawn from the antecubital vein of seated participants, with minimal tourniquet use. Specimens were collected in siliconized vacuum glass tubes containing a 1/10 volume of 3.8% trisodium citrate for blood glucose, and no additives for lipids. Tubes were centrifuged at 3000g for 15 minutes at room temperature. Serum samples were stored at 4 °C in refrigerated containers if the analysis was to be performed within a few days. Otherwise, the samples were frozen until analysis. Plasma samples were frozen as rapidly as possible to −80 °C for storage, until laboratory examination could be performed.

Total cholesterol was measured using a commercially available enzymatic method (Wako, Osaka, Japan; interassay coefficient of variation (CV): 1.5%). Blood glucose was measured via a commercially available enzymatic method (Kanto Chemistry, Tokyo, Japan; interassay CV: 1.9%). In this study, blood samples from 5547 (45.0%) participants were collected after overnight fasting. Diabetic participants were defined as those with currently treated diabetes, plasma glucose ≥126 mg/dl after an overnight fast, or casual blood glucose ≥200 mg/dl. Participants were asked to indicate whether they were current smokers or not.

### Follow-up

Repeat examinations (part of the national mass-screening program) were used to follow most participants every year. Those examined were asked whether they had experienced an MI after enrolling. Participants who did not come to an appointed screening examination were contacted by mail or phone. Public health nurses visited the participants to obtain pertinent information when necessary. In this study 100% of the participants were contacted. Those with a history of MI were asked where (in which hospital) they had been treated, and the date of medical diagnosis. Medical records at hospitals in the study area were also consulted to determine if these participants had been treated. If an incident was suspected, pertinent electrocardiograms were obtained in accordance with the law for diagnostic identification of MI. The medical records of all the suspected cases were obtained during follow-up. Diagnoses were determined independently by a diagnosis committee composed of 1 radiologist, 1 neurologist, and 2 cardiologists. A diagnosis of MI was determined by using the criteria of the World Health Organization Multinational Monitoring of Trends and Determinants in Cardiovascular Disease (MONICA) Project, a multinational collaborative project to monitor coronary events from the mid-1980s to the mid-1990s.^21^ Participants who met the MONICA criteria for a nonfatal or fatal “definite myocardial infarction” or “possible myocardial infarction” were defined as MI cases.

### Statistical analysis

Statistical analyses were performed using SPSS for Windows, version 11.5 (SPSS Inc, Japan). Data obtained for men and women were analyzed separately, and a Cox proportional hazards model was used to calculate the 10-year absolute risk of MI for each risk profile. Age, systolic blood pressure, total cholesterol, diabetes status, and current smoking status were entered into the proportional hazards model as explanatory variables. In the model, age, systolic blood pressure, and total cholesterol were treated as continuous variables, and diabetes status and smoking status were treated as dichotomous variables. The survival probability S(T:X) of a person with a risk X at time T was represented as S(T:X) = ([S_0_(T)]^exp(^*^B^*^X)^)^exp(^*^B^*^(X−Xm))^, where S_0_(T) is the survival probability corresponding to the standard hazard, *B* is the regression coefficient, and X_m_ is the population mean of risk X. The 10-year absolute risk of a person with risk X was thus defined as 1-S(10:X).^17^ The 10-year absolute risk of a person with multiple risk factors was calculated in a similar manner. For example, where X, Y, and Z are the risk factors, S(T:X,Y,Z) = [S_0_(T)]^exp(^*^B^*^X*^^B^^(X−Xm)+^*^B^*^Y*^^B^^(Y−Ym)+^*^B^*^Z*^^B^^(Z−Zm))^. Risk charts were created for both sexes based on calculations of the absolute risk associated with a quintet of conventional cardiovascular risk factors: age, smoking status, diabetes status, systolic blood pressure, and serum total cholesterol. Age was grouped into 5 categories: under 40, 40–49, 50–59, 60–69, and 70 years or older. Systolic blood pressure was grouped into 5 categories: less than 120, 120–139, 140–159, 160–179, and 180 mm Hg or higher. Total cholesterol was grouped into 6 categories: less than 180, 180–199, 200–219, 220–239, 240–259, and 260 mg/dl or higher. The other risk factors were treated as dichotomous variables. In total, the charts show absolute risks for 1200 combinations of risk factors. The risk charts were color-coded so that users could easily estimate their probability of a future MI.

### Ethical issues

The study design and procedures were approved by each community government, and by the Ethical Committee of Epidemiologic Research at Jichi Medical University. Written informed consent to participate in the study was obtained individually from each respondent to the mass screening.

## RESULTS

The baseline characteristics of study participants are shown in Table 1. The mean follow-up period (± SD) was 10.9 ± 2.25 (10.8 ± 2.44 years in men and 11.0 ± 2.12 years in women). Because the cohort is based in rural communities, the participants were older (mean age 55.0 years old in men and 55.3 in women) and more were female (60.8%), as compared with the Japanese general population. Ninety-two cases (64 men and 28 women) of MI were identified during follow-up. The overall crude incidence rate was 1.23/1000 person-years in men and 0.34/1000 person-years in women. After adjustment for age by the direct method, the incidence rate was 0.83/1000 person-years in men and 0.31/1000 person-years in women.^22^ The results of the proportional hazards analysis, upon which the absolute risk calculations were based, are shown in Table 2.

**Table 1.** Baseline characteristics of participants

	Men		Women	
Number of participants at baseline	4829	(100)	7494	(100.0)
Mean length of follow-up, years (SD)	10.8	(2.4)	11.0	(2.1)
Mean age at baseline, years (SD)	55.0	(12.0)	55.3	(11.3)
Mean body mass index, kg/m^2^ (SD)	23.0	(2.9)	23.2	(3.2)
Current smokers	2248	(46.6)	380	(5.1)
Mean total cholesterol, mg/dl (SD)	184.9	(34.2)	196.7	(34.8)
Mean systolic blood pressure, mm Hg (SD)	131.4	(20.5)	128.2	(21.0)
With diabetes	249	(5.2)	198	(2.6)
Cumulative incidence of myocardial infarction	64	(1.3)	28	(0.4)

Data are expressed as number (%) unless otherwise indicated.

SD: standard deviation

Diabetes: those with currently treated diabetes, plasma glucose ≥126 mg/dl after an overnight fast, or casual blood glucose ≥200 mg/dl.

**Table 2.** Hazard ratio for myocardial infarction for each risk factor in men and women

Sex	Variable	HR	95% CI	*P*
Men	Age (1 yr)	1.087	1.056–1.120	<0.001
	SBP (1 mm Hg)	1.016	1.004–1.027	0.006
	T-chol (1 mg/dl)	1.013	1.005–1.020	0.001
	Diabetes	0.791	0.245–2.553	0.695
	Current smoking	2.657	1.524–4.631	0.001
Women	Age (1 yr)	1.106	1.044–1.171	0.001
	SBP (1 mm Hg)	1.023	1.004–1.041	0.015
	T-chol (1 mg/dl)	1.009	0.996–1.021	0.168
	Diabetes	4.372	1.454–13.150	0.009
	Current smoking	3.047	0.704–13.200	0.136

HR: Hazard ratio

95% CI: 95% confidence interval

**Figure 1.**
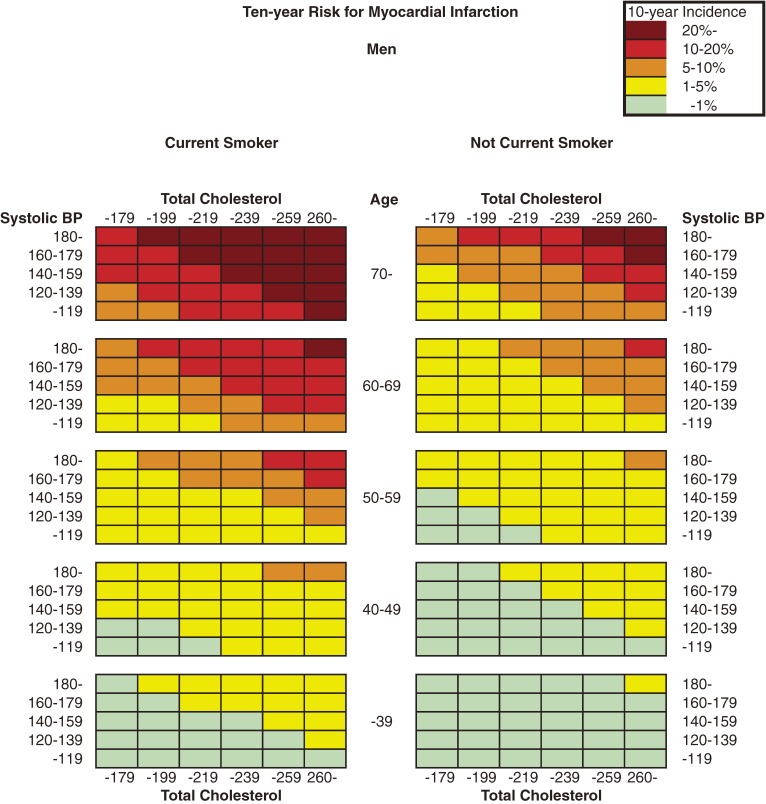
Chart showing 10-year risk for myocardial infarction in men

**Figure 2.**
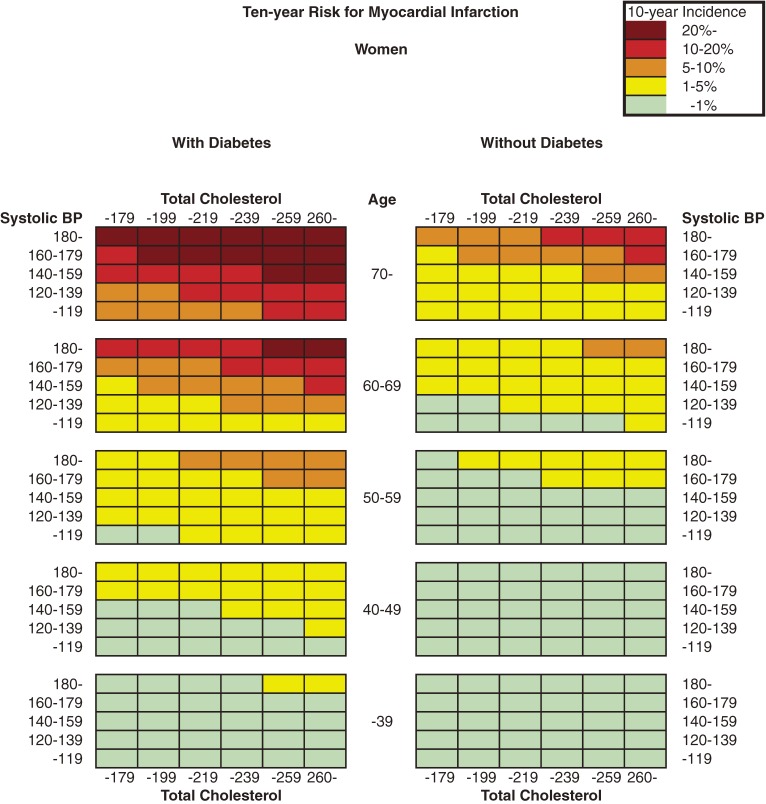
Chart showing 10-year risk for myocardial infarction in women

Figure 1 and Figure 2 show the color-coded 10-year absolute risk of MI for each combination of risk factors. Diabetes in men and current smoking status in women were not included in the risk estimations because these 2 factors were not significantly associated with MI incidence, as shown in Table 2. The charts were separated into 2 tables according to sex, and each was then subdivided further according to diabetes status and smoking status. Risk can be estimated by matching the person’s age to the nearest age grouping, total cholesterol level to the corresponding range, and blood pressure to the nearest multiple of 20 mm Hg.

The risk of MI increased as systolic blood pressure rose, which is clearly indicated by the data shown in the Figures. Similarly, the risk of MI increased with elevation of total cholesterol level. Current smoking in men and diabetes in women also increased the 10-year risk of MI.

## DISCUSSION

We developed simple risk charts by using data on a specific cardiovascular event (MI) from a Japanese cohort study. The charts illustrate the 10-year absolute risk of MI associated with sex, age, smoking status, diabetes status, total cholesterol, and blood pressure. The charts should prove to be convenient and beneficial tools for health education. They can be used by both health professionals and individual patients, without the need for cumbersome calculations.

The data upon which the charts are based are derived from a Japanese community-based cardiovascular cohort study. The charts are thus more suitable for Japanese populations than charts based on non-Japanese populations, which possess very different genetic and environmental characteristics. The problems of applying the data from the Framingham study to populations other than North American white populations have been frequently described.^10^^,^^23^^–^^29^ The major problem with using foreign risk charts is that incidences of cardiovascular events differ among populations. To address this problem in Europe, a multinational cardiovascular cohort study was conducted, and risk charts for MI were developed that were based on original incidence data from European populations.

In Japan, too, cardiovascular risk charts were created by using data from NIPPON DATA80, a 19-year follow-up study of Japanese populations.^16^^,^^17^ The pattern of relations between conventional risk factors and coronary heart disease (color distribution) in the charts from the NIPPON DATA80 is largely similar to that shown in our charts. In both risk charts, most conventional risk factors increase the probability of coronary heart disease. The relations agree with the results of other cohort studies conducted in Japan.^3^ However, the NIPPON DATA80 captured cardiovascular deaths, and in that respect, differs from the JMS cohort study, where the outcome of interest was cardiovascular events. Somewhat surprisingly, the crude rate of coronary heart disease (CHD) in NIPPON DATA80 was higher than the incidence of MI in our study (0.74 vs. 0.68 per 1000 person-years),^16^ even though the outcome of interest was cardiovascular death in the NIPPON DATA80. This result is probably due to the higher prevalences of risk factors in the NIPPON DATA80 cohort. Average systolic blood pressure in NIPPON DATA80, for example, is substantially higher than that in our study (men: 138.4 vs. 131.4 mm Hg; women: 133.9 vs. 128.2 mm Hg).^16^

However, despite the lower crude rate of MI in our study, the estimated 10-year risks of MI in our charts are mostly higher than the CHD risks in the NIPPON DATA80 charts. These seemingly contradictory results are largely attributable to differences between the 2 studies in hazard ratios for MI for some risk factors. In this study, as compared to the respective hazard ratios from NIPPON DATA80, the hazard ratio for MI for systolic blood pressure in women is six times as high in the present study, and those for female total cholesterol and male smoking were twice as high.^17^

The First and Second Joint Task Force of European and other Societies on Coronary Prevention adopted colored coronary risk charts and recommended that a 10-year absolute risk for MI of 20% be used as a threshold for intensified risk factor intervention, including lifestyle modification and selective use of proven drug therapies.^30^ Although no such threshold of absolute risk is specified in the guidelines for the primary prevention of ischemic heart disease issued by the Japanese Circulation Society Joint Working Group (JCS 2006), the guidelines do recommend intervention for any modifiable factors known to be risks for coronary heart disease.^3^ The present charts will assist health professionals in identifying high-risk individuals who require intervention to maintain good health. Indeed, these individuals themselves can also obtain beneficial information from the charts, such as the degree to which their MI risk decreases when they improve their risk factors.

There are some limitations in this study. First, this study focused only on MI and did not include angina, due to the difficulties in obtaining a definite diagnosis of the condition. Thus, the absolute risks calculated in this study are lower than would be if the risks of “coronary heart disease” had been considered in a broad sense. Second, this study did address stroke—the most prevalent cardiovascular event in Japan. The JMS Cohort Study did capture stroke events. Therefore, separate charts for estimating stroke risks based on the cohort data could and should be developed in the future. Third, most of the participants in the JMS Cohort Study were rural residents of retirement age or older. The incidence of MI in a group of participants can vary depending on living environment (rural vs. urban) and age studied (old vs. young populations). Use of the charts with different populations should be done with care.
